# Obituary: Odile Bain (28/04/1939–16/10/2012)

**DOI:** 10.1051/parasite/2013022

**Published:** 2013-06-19

**Authors:** Coralie Martin


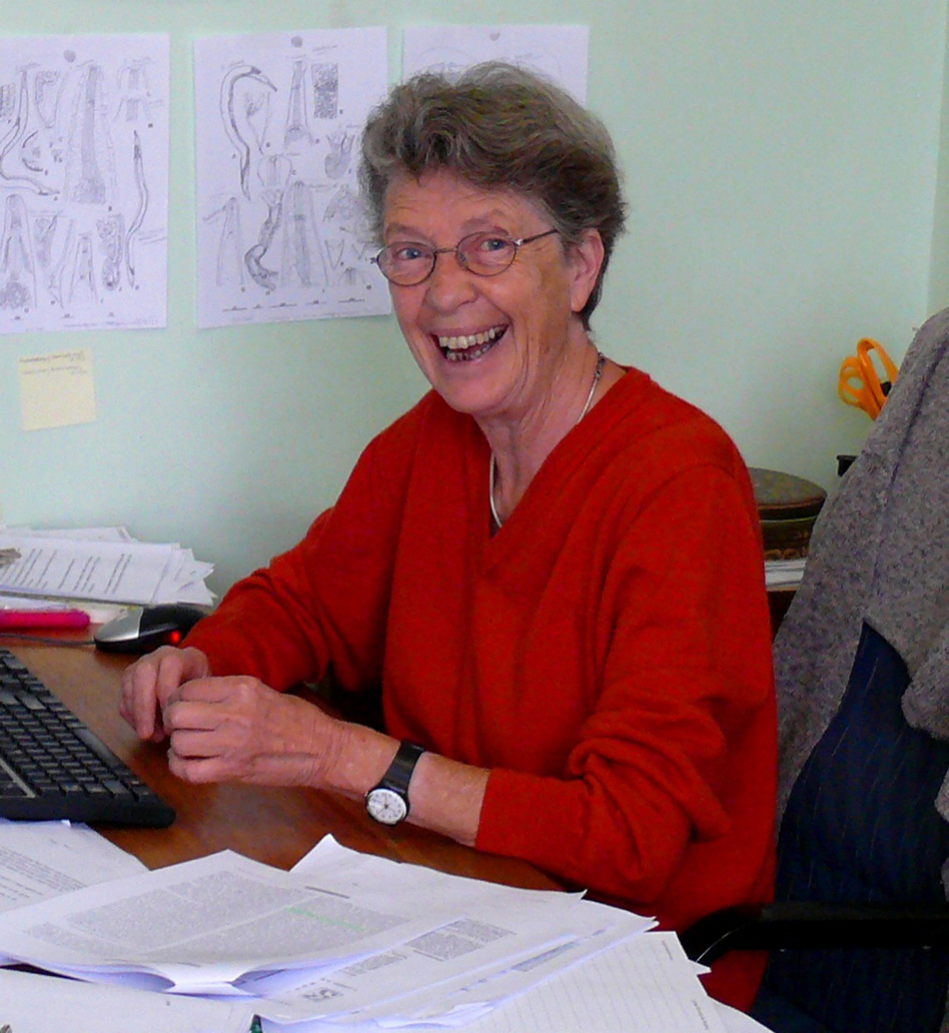


Odile Bain was born on April 28, 1939 in Dalat (VietNam) where her father, a military officer, was based at the time. Her high school days were spent in Dakar (Senegal) and in 1960 she graduated from the Faculty of Biology in Rennes (France). After a short stay at the Faculty as a teaching assistant, she moved to the Muséum National d’Histoire Naturelle in Paris (France) in September 1964, where she joined the Laboratory “Zoologie des Vers”. The laboratory, nowadays a veritable lesson in the history of Parasitology, had been founded by Prof. Alain G. Chabaud in 1961, and gathered specialists of different groups of “worms” and Protozoa. Prof. Chabaud supervised several teams of research on parasitic Nematodes, Trematodes, Cestodes and Protozoa. Many stories are told about this laboratory at the time of its original location in rue Cuvier and its present location in rue Buffon. Most of all, it was famous for its unbridled sense of welcome, good food and outstanding science.

Here, Odile rose through the ranks of the National Centre for Scientific Research (CNRS), built an extraordinary career in Parasitology and was finally awarded the title of Director of Research (DR) of Exceptional Class.

In 1988, when presenting her scientific work to qualify for a rating as DR2 CNRS, Odile wrote the following: “My career is simple: it is carried out entirely at the Natural History Museum under the direction of Prof. A.G. Chabaud. My specialization is easy to define: it is the systematic and biology of filariae”. Parasitologist, morphologist, passionate about biology and microscopy, she – despite her modesty – proceeded to become the world authority in her chosen field. Over time, she expanded the scope of her studies, but while her research interests were numerous and varied, the systematics and biology of filarial worms as well as the phyletic relationships between their different lineages and with other groups of nematodes remained at the basis of her activities. During her career she extended her interest to the vectors and the transmission of filariae; her third major field of study was the interactions between the filariae and their vertebrate hosts, and more recently with the endobacteria *Wolbachia*. This orientation opened new avenues in the study of filarial diseases, chemotherapy and immunology. One of the major scientific advances in the study of filariasis was brought about by Odile’s development of the first experimental model of filariasis in mice, a model now used worldwide. To summarize her long and productive career in figures: during her almost 50 years in research, Odile published more than 360 scientific articles. She held 30 years of successive contracts with the World Health Organisation, Edna McConnell Clark Foundation and the European Union. She was awarded the bronze medal of the CNRS in 1974 and received the Prize of Zoology of the Foulon Academy of Sciences in 1984.

Impressive as they are, these figures nevertheless fail to adequately reflect the true impact of her life’s work and the vast contribution made by her to her field of expertise. Odile was passionate about research and had a genuine modesty about her work. She considered herself privileged to work on research topics most often fundamental. It was her firm belief that a scientist must first and foremost share their results with the scientific community. In 1996, in her application for a DR1 rating, she wrote: “I have a strong interest in the review of the French Society of Parasitology, Parasite, which follows since 1994 the journal “Annales de Parasitologie Humaine et Comparée”. I think it is very important to maintain a certain degree of freedom and timeliness and we need to support and develop this tool”. It was an interest and an association she maintained throughout her career.

Another subject close to her heart was the notion of networking; in her opinion research was not a national issue. She was a convinced European in every sense and particularly in the field of research. This state of mind, her belonging to a strong European consortium and her many international contacts enabled her and her team to accommodate a multitude of researchers from all continents and to participate in numerous transnational projects. Scientifically, it was exciting and productive to work in such an environment; culturally, it was extremely enriching.

Odile was not only an outstanding – and unconventional – scientist but also a woman of great generosity with real human qualities. She requested a true daily involvement in research from her team and was demanding regarding the quality of the work, but in return she was always present and available to support the completion of projects and articles. Odile was open, smiling, exuberant in her passion for science, confident and respectful of her colleagues – whatever their status; her motivation were knowledge and the pleasure of discovery. It is these qualities that marked her as one of the great naturalists who contributed to the reputation of the Museum, and that made it a priviledge to be associated with her. Her research has inspired many scientists around the world, who strive to preserve and advance the knowledge she shared so freely with the scientific community. We all feel this loss deeply and will remember Odile, smiling and always ready to take on new scientific challenges.

